# Synthesis of millimeter-scale ZIF-8 single crystals and their reversible crystal structure changes

**DOI:** 10.1080/14686996.2023.2292485

**Published:** 2024-01-19

**Authors:** Azhar Alowasheeir, Nagy L. Torad, Toru Asahi, Saad M. Alshehri, Tansir Ahamad, Yoshio Bando, Miharu Eguchi, Yusuke Yamauchi, Yukana Terasawa, Minsu Han

**Affiliations:** a Department of Materials Process Engineering, Graduate School of Engineering, Nagoya University, Nagoya, Japan; b Chemistry Department, Faculty of Science, Tanta University, Tanta, Egypt; c Department of Chemistry, Khalifa University, Abu Dhabi, United Arab Emirates; d Advanced Materials Chemistry Center (AMCC), Khalifa University, Abu Dhabi, United Arab Emirates; e School of Advanced Science and Engineering, Waseda University, Shinjuku-ku, Tokyo, Japan; f Chemistry Department, College of Science, King Saud University, Riyadh, Saudi Arabia; g Australian Institute for Innovative Materials, University of Wollongong, North Wollongong, New South Wales, Australia; h Australian Institute for Bioengineering and Nanotechnology (AIBN), The University of Queensland, Brisbane, Queensland, Australia; i Kagami Memorial Research Institute for Materials Science and Technology, Waseda University, Shinjuku-ku, Tokyo, Japan; j Faculty of Advanced Science and Technology, Kumamoto University, Kumamoto-shi, Kumamoto, Japan

**Keywords:** Millimeter-scale ZIF-8, solvothermal, single crystal, single crystal X-ray diffraction

## Abstract

Among various metal-organic frameworks (MOFs), the zeolitic imidazole framework (ZIF), constructed by the regular arrangement of 2-methylimidazole and metal ions, has garnered significant attention due to its distinctive crystals and pore structures. Variations in the sizes and shapes of ZIF crystals have been reported by changing the synthesis parameters, such as the molar ratios of organic ligands to metal ions, choice of solvents, and temperatures. Nonetheless, the giant ZIF-8 single crystals beyond the typical range have rarely been reported. Herein, we present the synthesis of millimeter-scale single crystal ZIF-8 using the solvothermal method in N,N-diethylformamide. The resulting 1-mm single crystal is carefully characterized through N_2_ adsorption-desorption isotherms, scanning electron microscopy, and other analytical techniques. Additionally, single-crystal X-ray diffraction is employed to comprehensively investigate the framework’s mobility at various temperatures.

## IMPACT STATEMENT

Millimeter-sized ZIF-8 single crystals were synthesized using the solvothermal method. These crystals exhibit a notable BET surface area of 1681 m^2^∙g^−1^ and demonstrate a reversible change in their crystal structure.

## GRAPHICAL ABSTRACT

**Figure:**
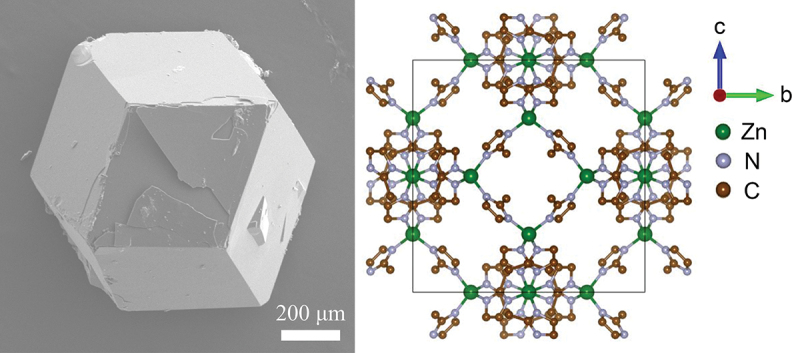

## 1. Introduction

A zeolitic imidazolate framework (ZIF) belongs to the subclass of metal-organic frameworks (MOFs) and represents a porous crystalline structure. The ZIFs are constructed from tetrahedral units, where a bivalent metal cation, like Zn^2+^ or Co^2+^, is bonded to four imidazolate units (M(Im)_4_; where M represents a metal cation, and Im represents an imidazolate unit) [1]. The bonding angle between the imidazolate units and the bivalent metal cation (M-Im-M) closely resembles the Si-O-Si angle (145°), leading to the formation of various ZIFs with zeolite-like topological structures [2]. ZIF-8, which is constructed using 2-methylimidazole units and Zn ions, has received significant attention due to its appealing structural properties and numerous potential applications [3–8]. Unique structural characteristics including consistent pore structures and substantial surface area, and a straightforward synthetic approach have made ZIF-8 a long-standing focal point of attention [9]. The topology of ZIF-8 consists of cages with a size of 1.16 nm connected through six-membered windows measuring 0.34 nm [10,11].

The size, shape, and pore structure of ZIF crystals have been widely recognized to be influenced by synthetic parameters such as the molar ratios of organic ligands to metal ions [12,13], solvents [14], additives [15], and reaction temperatures [16]. However, these reaction parameters did not exhibit consistent effects on the synthesis of ZIF-8 and, in some cases, yielded contrary outcomes depending on the system, suggesting the involvement of multiple mechanisms in ZIF-8 synthesis. This multifaceted nature explains the scarcity of reports on the synthesis of large-scale ZIF-8 single crystals (SCs-ZIF-8). Only a limited number of reports have provided unequivocal evidence regarding the synthesis of large-scale SC-ZIF-8, and even in those instances, the crystal shapes were not flawless [16,17]. Ladewig *et al*. successfully synthesized SCs-ZIF-8 with sizes of up to 1 mm using a solvothermal reaction in methanol, with sodium formate serving as a reaction modulator [18]. The SC-ZIF-8 exhibited outstanding performance in gas adsorption and separation, benefiting selectivity based on its constant pore structure [19]. The surface area is a vital property for various applications due to its association with the active sites interacting with reactive species. Notably, N,N-dimethylformamide (DMF) and methanol have been the frequently employed solvents in ZIF-8 synthesis, primarily aiming for increased surface areas [20], likely due to the relatively low dielectric constant, dipole moment, and hydrogen bond donation of organic solvents [14,21]. The crystal structure of MOFs can influence their pore structures and chemical properties and is subject to reversible control through temperature, pressure, and additives [22–26]. Single crystal X-ray diffraction (SC-XRD) pattern is a powerful tool for revealing a slight increase in the lattice parameters of SC-ZIF-8 with rising temperatures within the range of 100–298 K [27]. The expansion of the nanopore and unit cell on SC-ZIF-8 was also confirmed under high-pressure conditions [28,29].

This study aims to synthesize large-sized SC-ZIF-8 using the solvothermal method with N,N-diethylformamide (DEF) as the solvent, optimizing a procedure from the literature [10]. A minute quantity of HNO_3_ solution is employed to regulate the seed formation rate at the onset of the synthesis. The porosity and chemical structure of the SC-ZIF-8 are analyzed through N_2_ adsorption-desorption isotherms and Fourier-transform infrared (FTIR) spectroscopy. Furthermore, it serves as a model material for investigating framework mobility within the range of 100–500 K, utilizing SC-XRD patterns.

## 2. Experimental Section

### 2.1 Materials

Zinc nitrate hexahydrate (98%, Wako), N,N-diethylformamide (DEF, Tokyo Chemical Industry), N,N-dimethylformamide (DMF, Nacali), nitric acid (S.G. = 1.42, 70%, Nacali), and 2-methylimidazole (Wako) were commercially purchased and utilized without further treatment.

### 2.2 Synthesis of SC-ZIF-8

In a 20 mL screw-top vial, 350 mg of Zn(NO_3_)_2_·6 H_2_O and 200 mg of 2-methylimidazole were introduced and dissolved in 15 mL of DEF. 60 μL of 70% HNO_3_ solution was carefully added to the mixture using a Pasteur pipette, and complete dissolution was achieved by sonication. The vial was then sealed and placed in an oven at 120°C for 72 h. The resulting ZIF-8 crystals were collected through filtration and washed with DMF. Subsequently, the crystals were stored in DMF at room temperature until further use.

### 2.3 Characterization

All crystal structures were determined using the SC-XRD XtaLAB Synergy-S (Rigaku, Oxford Diffraction, Japan) (Cu K α (*λ* = 1.54184)), equipped with a HyPix-6000HE Hybrid Photon Counting detector and dual Mo and Cu microfocus sealed tube. Approximately 0.1 mm of crystal was mounted on a glass rod and cooled to 100 K. The investigation of framework mobility was conducted on the same crystal using SC-XRD. XRD patterns were recorded while maintaining temperatures ranging from 100 to 500 K with a 100 K interval. The temperature was ramped up or down at a rate of 100 K∙h^−1^, and the crystal was allowed to equilibrate at the target temperature for 30 min before each XRD measurement. Three samples were employed to demonstrate the reproducibility of SC-XRD patterns at different temperatures. The raw data were processed using CrysAlisPro [30]. The initial structure was solved using direct methods with SHELXT [31], and the structure was subsequently refined by least-squares using SHELXL2018. All atoms, excluding hydrogen atoms, were refined with U_ij_ values. FTIR spectroscopy was employed to identify the chemical constituents in the samples. The samples were mixed with potassium bromide (KBr) and pressed into pellets. FTIR spectra were recorded at room temperature using the Thermo Scientific Nicolet 4700 instrument (Thermo Fisher Scientific, USA). Thermogravimetric (TG) analysis of the samples was performed using a Rigaku TG8120 instrument (Rigaku, Japan), heating the samples from room temperature to 1000°C under nitrogen at a fixed heating rate of 10°C min^−1^. N_2_ adsorption-desorption isotherms of the samples were measured on a Quantachrome Autosorb gas sorption system (Anton Paar, Austria) at 77 K. Before the measurement, the samples were treated at 150°C for 24 h. The morphological characterization of the samples was conducted using a Hitachi SU8000 scanning electron microscope (SEM) operating at an accelerating voltage of 5 kV.

## 3. Results and discussion

The deprotonation rate of 2-methylimidazole plays a crucial role in ZIF-8 synthesis, and various factors, such as solvents, pHs, and additives, have been employed to control this rate [14,32–34]. In the synthesis of SC-ZIF-8, the addition of a minute quantity (60 μL) of HNO_3_ solution is pivotal. The introduction of acid suppresses deprotonation, consequently limiting the initial seed formation and facilitating the formation of large SC-ZIF-8. It is worth noting that SC-ZIF-8 cannot be obtained when other acids, such as H_2_SO_4_ or HCl, are employed.

**Figure 1.**
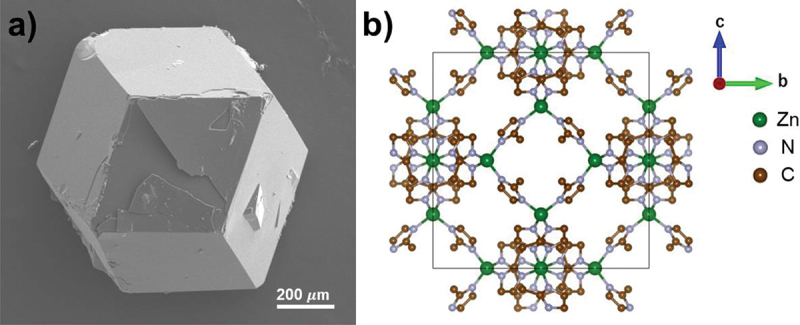
Synthesized rhombic dodecahedron-shaped SC-ZIF-8. (a) SEM image of the millimeter-scale SC-ZIF-8 viewed along [110] axis. (b) Crystal structure obtained by SC-XRD [100] of the SC-ZIF-8. The hydrogen atoms in the structure have been removed for the sake of clarity.

**Figure 2.**
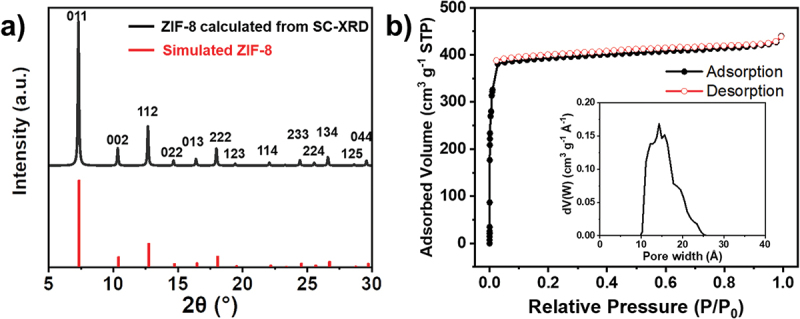
(a) XRD pattern of the SC-ZIF-8 calculated from SC-XRD and simulated ZIF-8. (b) N_2_ adsorption-desorption isotherms of the SC-ZIF-8 (inset: NLDFT pore size distribution).

Figures 1(a) and **S1** show the brown, transparent, crack-free SC-ZIF-8, which reaches a size of 1 mm and exhibits a rhombic dodecahedron morphology. Some crystals also display cubic morphology (Figure S1b,c), indicating the presence of unevolved crystals even after a 72-hour reaction. These cubic morphologies are intermediates and they gradually evolve into truncated edges [16,35,36]. The XRD pattern of SC-ZIF-8 (Figure 2(a)) shows sharp and intense peaks, indicating a high-quality crystal [37,38]. The overall XRD pattern matches well with the simulated pattern (JCPDS 00-062-1030).

Previous studies have extensively investigated the surface area modulation of ZIF-8 by altering the synthesis conditions [13,14,39]. Since several factors simultaneously influence porous properties, including surface area, the specific effect of one factor on these properties has not been clearly identified. The N_2_ adsorption-desorption isotherms of SC-ZIF-8 were measured at 77 K, exhibiting a typical type I isotherm characteristics of microporous materials [40] (Figure 2(b)). The corresponding Brunauer – Emmett–Teller (BET) surface area is calculated to be 1681 m^2^∙g^−1^, and the pore volume is 0.62 cm^3^∙g^−1^. This value represents the highest surface area reported in recent SC-ZIF-8 studies (Table S1). The pore size distribution curve calculated by the non-local density functional theory (NLDFT) reveals that the pore width of SC-ZIF-8 ranges between 10 and 25 Å, indicating that SC-ZIF-8 is primarily composed of micropores.

The SC-XRD patterns of the rhombic dodecahedron-shaped SC-ZIF-8 at 100 K confirm its crystal structure in the *I*$\overset{ˉ}{4}$3*m* space group, with a lattice parameter a of 16.8941(3) Å (Table 1 and Figure 1(b)). To investigate the framework mobility of the crystal, temperature variations were applied during SC-XRD. Temperatures were increased in 100 K intervals from 100 to 500 K, with a temperature rise rate was 100 K∙h^−1^. The crystal was equilibrated at each temperature for 30 min before data collection. After the structural refinement, accessible voids large enough to contain a solvent are found in every structure. Conversely, assuming that the solvent mask (with DEF used in the synthesis) exists in the crystal, the structure refinement yields a reasonable structure. Consequently, the resulting structure is determined as a ZIF-8 crystal containing a disordered DEF solvent mask. Up to 300 K, the SC-XRD data of the crystal exhibit similarities to previously reported ZIF-8 structures [27]. Despite an increase in lattice parameter to 17.1048(4) Å, the original *I*$\overset{ˉ}{4}$3*m* space group is maintained as the temperature rises. Notably, the Zn1-N2 bond length increases from 1.987(3) Å at 100 K to 1.996(3) Å at 500 K with increasing temperature (Table S4). In addition, the nitrogen-to-nitrogen distance (N2’’⋯N2’’’) between adjacent 2-methylimidazole linkers (Figure S2) increases from 3.242(6) to 3.263(5) Å, while the N2’’-Zn1-N2’’’ angle increases from 109.39(16)° to 109.67(13)°. Conversely, the carbon-to-carbon distance (C5⋯C5’) between adjacent 2-methylimidazole linkers remains a minimum distance of 0.427 nm above 300 K, indicating no interaction between these atoms [27]. Generally, the cell volume expands from 4821.8(3) to 5004.4(4) Å^3^ with increasing temperature, which aligns with observations under high-pressure conditions [28,29].

**Table 1.** Summary of SC-XRD data of the SC-ZIF-8 collected with increasing temperature.

Temperature (K)	100(2)	200(2)	300(2)	400(2)	500(2)
Crystal size (mm)	0.1 × 0.1 × 0.1				
Chemical formula	C_2_H_2.50_NZn_0.25_				
Crystal system	Cubic				
Space group	*I*$\overset{ˉ}{4}$3*m*				
*a* (Å)	16.8941(3)	16.9524(2)	17.0307(4)	17.0865(2)	17.1048(4)
*V* (Å^3^)	4821.8(3)	4871.85(17)	4939.7(3)	4988.38(18)	5004.4(4)
*Z*	48	48	48	48	48
R[F^2^ > 2σ(F^2^)]	0.0280	0.0226	0.0247	0.0221	0.0220
ωR (F^2^)	0.0748	0.0569	0.0620	0.0546	0.0461
S	1.026	1.080	1.040	0.994	0.948

**Figure 3.**
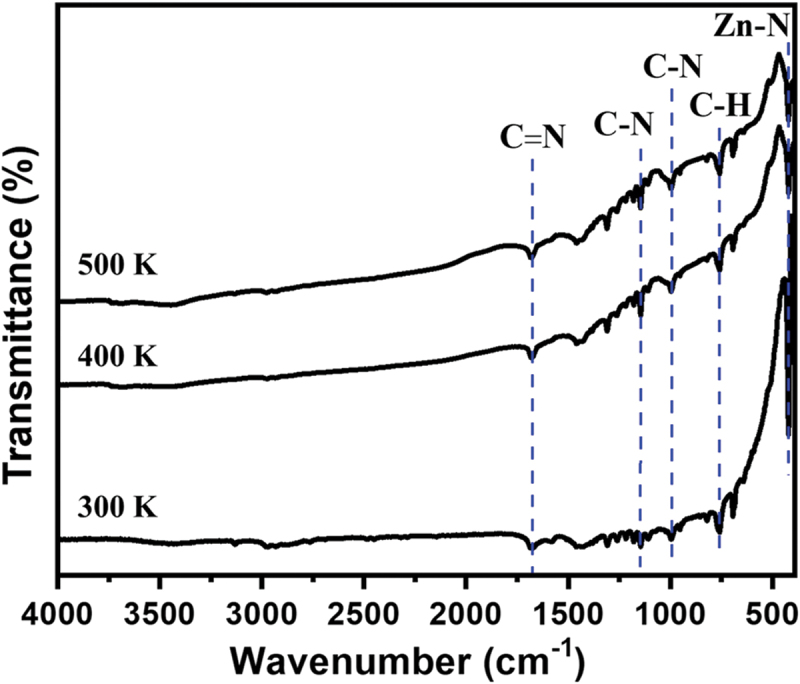
FTIR spectra of the SC-ZIF-8 at 300, 400, and 500 K.

**Figure 4.**
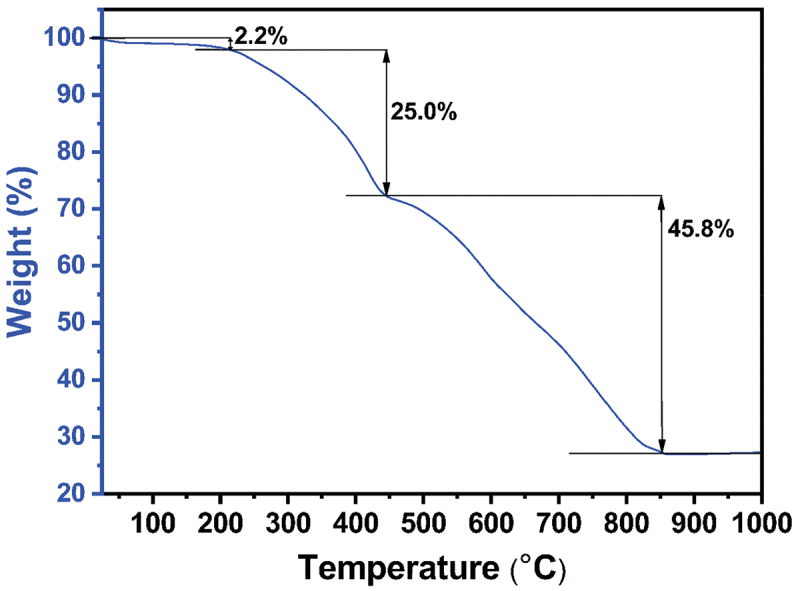
TG curve for SC-ZIF-8 under nitrogen gas flow.

Fourier transform infrared spectroscopy (FTIR) spectra of the crystal at 300, 400, and 500 K confirm the absence of changes in chemical functional groups, such as the Zn-N stretching band and the in/out-of-plane bending of 2-methylimidazole [41,42] (Figure 3). This observation confirms that there are no noticeable changes as the temperature increases up to 500 K. The thermal stability of SC-ZIF-8 was assessed through thermogravimetric (TG) analysis, revealing a three-step weight loss process (Figure 4). Initially, a minor weight reduction occurs up to 220°C, constituting a 2.2% weight loss attributed to the removal of water molecules from the surface of SC-ZIF-8 [43]. The second weight reduction, a significant decrease persisting until 445°C, results in a 25.0% weight loss, reflecting the removal of DEF. Similar substantial weight reductions between 200 and 400°C have been observed in other SC-MOFs containing solvents [2], suggesting a challenge in the evaporation of internal solvents within MOF crystals. Therefore, based on this data, the solvents can persist until 445°C (718 K), aligning with the SC-XRD result indicating the presence of DEF in the ZIF-8 crystal structure (Table 1). Beyond 445°C, the third weight reduction occurs, continuing until reaching 860°C. During this phase, ZIF-8 undergoes decomposition, eliminating all organic components and Zn content.

For further investigation, the collected data obtained by SC-XRD with decreasing temperatures (400, 300, 200, and 100 K) using the same crystal after the measurement at 500 K shown in Table 1 is presented in Table 2. These structures were also determined as a ZIF-8 crystal containing a disordered DEF solvent mask. As the temperature gradually decreases to 100 K, the lattice parameter decreases from 17.1048(4) Å at 500 K to 16.9466(3) Å at 100 K. The bond length of Zn1-N2 decreases with decreasing temperature (Table S5). However, up to 300 K, the N2’’⋯N2’’’ distance between adjacent 2-methylimidazole linkers remains relatively constant at about 0.325 nm. The C5⋯C5’ distance between adjacent 2-methylimidazole linkers increases to 4.319(7) Å at 100 K. Furthermore, the C3-N2’’-C4 angle in the 2-methylimidazole linkers also increases with decreasing temperature. With the temperature gradient, the cell volume decreases from 5004.4(4) Å^3^ at 500 K to 4866.8(3) Å^3^ at 100 K. These findings confirm that the structural changes observed in SC-ZIF-8 are reversible, and the SC-ZIF-8 reverts to its as-synthesized state.

**Table 2.** Summary of SC-XRD data of SC-ZIF-8 collected with decreasing temperature.

Temperature (K)	400(2)	300(2)	200(2)	100(2)
Crystal size (mm)	0.1 × 0.1 × 0.1			
Chemical formula	C_2_H_2.50_NZn_0.25_			
Crystal system	cubic			
Space group	*I*$\overset{ˉ}{4}$3*m*			
*a* (Å)	17.0523(3)	17.0074(4)	16.9827(2)	16.9466(3)
*V* (Å^3^)	4958.5(3)	4919.4(3)	4898.02(17)	4866.8(3)
Z	48	48	48	48
R[F^3^ > 2σ(F^3^)]	0.0273	0.0257	0.0171	0.0256
ωR (F^3^)	0.0700	0.0673	0.0410	0.0635
S	1.014	1.059	0.994	1.033

## 4. Conclusions

In summary, we successfully synthesized pure ZIF-8 single crystals with a size of 1 mm using DEF as the solvent *via* the solvothermal method at 120°C for 72 h. The obtained SC-ZIF-8 exhibits a high surface area of 1681 m^2^∙g^−1^ as determined by BET measurement. SC-XRD analysis reveals that the lattice parameter of SC-ZIF-8 exhibits temperature-dependent changes in the range of 100 to 500 K. These changes in lattice parameters can be attributed to variations in the bond lengths and angles of Zn-N and the 2-methylimidazole linker. The observed structural flexibility of SC-ZIF-8 suggests its potential suitability for various applications, including gas storage applications.

## Supplementary Material

Supplemental Material
