# Supplementary material for: Synthesis of millimeter-scale ZIF-8 single crystals and their reversible crystal structure changes
Source: Sci Technol Adv Mater. 2024 Jan 19;25(1):2292485. doi: 10.1080/14686996.2023.2292485 (PMC10802801; doi:10.1080/14686996.2023.2292485)
Supplement: Supplemental Material [file TSTA_A_2292485_SM7377.docx]

**Supplementary Information**

**Synthesis of millimeter-scale ZIF-8 single crystals and their reversible crystal structure changes**

Azhar Alowasheeir,^a^ Nagy L. Torad,^b,c,d^ Toru Asahi,^e^ Saad M. Alshehri,^f^ Tansir Ahamad,^f^ Yoshio Bando,^f,g^ Miharu Eguchi,^e,h^ Yusuke Yamauchi,^a,h^ Yukana Terasawa,^i,j*^ and Minsu Han^h*^

a Department of Materials Process Engineering, Graduate School of Engineering, Nagoya University, Nagoya 464–8603, Japan.

b Chemistry Department, Faculty of Science, Tanta University, Tanta, 31527 Egypt.

c Department of Chemistry and Advanced Materials Chemistry Center (AMCC), Khalifa University, P.O. Box 127788, Abu Dhabi, United Arab Emirates.

d Advanced Materials Chemistry Center (AMCC), Khalifa University, P.O. Box 127788, Abu Dhabi, United Arab Emirates

e School of Advanced Science and Engineering, Waseda University, 3-4-1, Okubo, Shinjuku-ku, Tokyo 169-8555, Japan.

f Chemistry Department, College of Science, King Saud University, P.O. Box 2455, Riyadh 11451, Saudi Arabia.

g Australian Institute for Innovative Materials, University of Wollongong, Squires Way, North Wollongong, New South Wales 2500, Australia.

h Australian Institute for Bioengineering and Nanotechnology (AIBN), The University of Queensland, Brisbane, Queensland 4072, Australia.

i Kagami Memorial Research Institute for Materials Science and Technology, Waseda University, 2-2 Wakamatsu-cho Shinjuku-ku, Tokyo-162-8480 Japan.

j Faculty of Advanced Science and Technology, Kumamoto University, Kumamoto-shi, Kumamoto, Japan.

*Corresponding author: [minsu.han@uq.edu.au](mailto:minsu.han@uq.edu.au); [terasawa@cs.kumamoto-u.ac.jp](mailto:tinkerbell@akane.waseda.jp)

**Supplementary Figures**


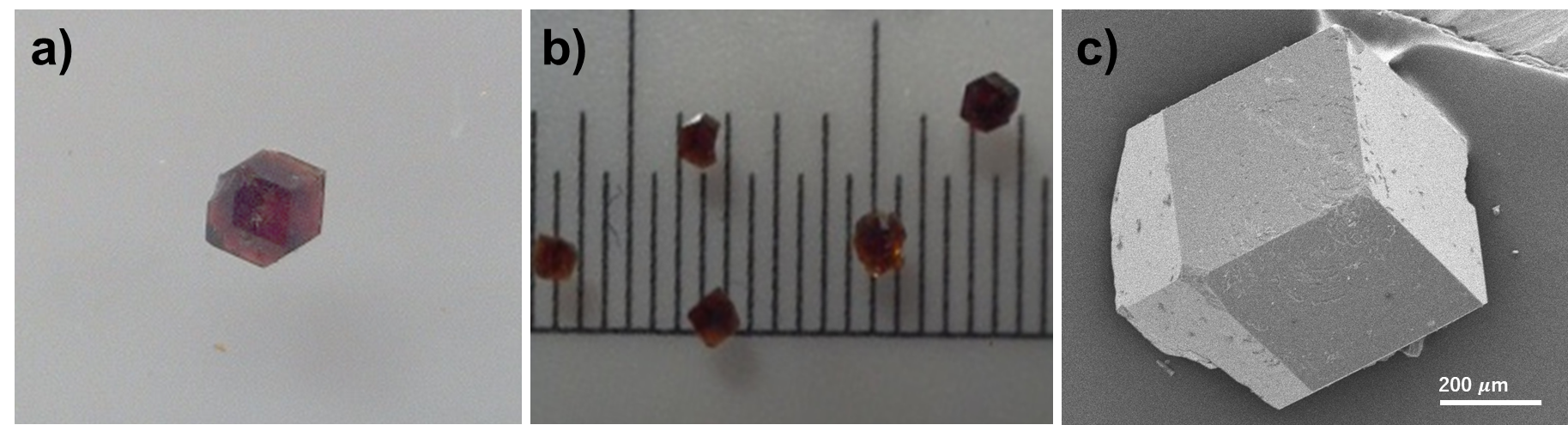


**Figure S1.** (a) As-prepared SC-ZIF-8 sample with a rhombic dodecahedron morphology. (b) Optical micrograph of millimeter-sized SCs-ZIF-8. (c) SEM image of the synthesized SC-ZIF-8 in cubic shape.


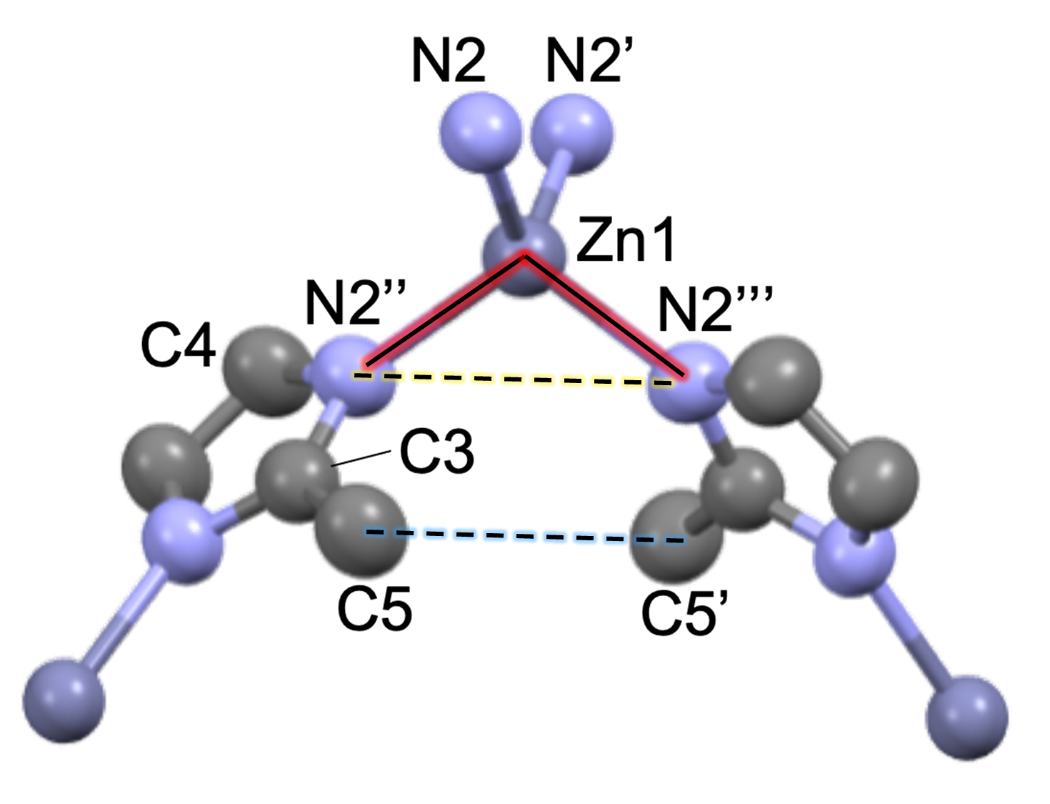


**Figure S2**. Assignment of nitrogen (dashed line, yellow), carbon (dashed line, blue), and angle of N-Zn-N (solid line, red) environments in ZIF-8.

**Supplementary Tables**

**Table S1**. Summary of the synthesis approach and properties of ZIF-8.

| Synthetic condition | | BET (m^2^.g^−1^) | Ref. |
| --- | --- | --- | --- |
| **Solvent** | **conditions** |  |  |
| DMF | 120^o^C, 24 h | 1080 | 1 |
| Methanol | 90^o^C, 24 h | 1490 | 2 |
| Methanol | 90^o^C, 24 h | 1560 | 3 |
| Methanol | 140^o^C, 24 h | - | 4 |
| DMF | 140^o^C, 24 h | 1630 | 5 |
| DEF | 120^o^C, 72 h | 1681 | This work |

**Table S2**. Complete data of SC-XRD of the SC-ZIF-8 (sample No.1) collected with increasing temperature.

| Temperature (K) | 100(2) | 200(2) | 300(2) | 400(2) | 500(2) |
| --- | --- | --- | --- | --- | --- |
| Chemical data | | | | | |
| Chemical formula | C_2_H_2.50_NZn_0.25_ | | | | |
| *M*_r_ | 56.89 | | | | |
| Crystal system | cubic | | | | |
| Space group | *I*$\bar{4}$3*m* | | | | |
| *a* (Å) | 16.8941(3) | 16.9524(2) | 17.0307(4) | 17.0865(2) | 17.1048(4) |
| *V* (Å^3^) | 4821.8(3) | 4871.85(17) | 4939.7(3) | 4988.38(18) | 5004.4(4) |
| *Z* | 48 | 48 | 48 | 48 | 48 |
| *μ* (mm^−1^) | 1.918 | 1.899 | 1.872 | 1.854 | 1.901 |
| crystal size (mm) | 0.1×0.1×0.1 | | | | |
| Data collection | | | | | |
| Diffractometer | XtaLAB Synergy-S (Rigaku) | | | | |
| Absorption correction | multi-scan (CrysAlisPro (Rigaku Oxford Diffraction, 2021)) | | | | |
| *T*_min_ | 0.64032 | 0.67413 | 0.75909 | 0.95110 | 0.64032 |
| *T*_max_ | 1.00000 | 1.00000 | 1.00000 | 1.00000 | 1.00000 |
| No. of measured, independent and observed [*I*>2*σ*(*I*)] reflections | 2734, 812, 766 | 2744, 826, 813 | 2784, 834, 805 | 2855, 843, 799 | 2836, 843, 727 |
| *R*_int_ | 0.0266 | 0.0259 | 0.0259 | 0.0233 | 0.0245 |
| (*sinθ*/*λ*)max (Å^-1^) | 0.615 | 0.614 | 0.614 | 0.614 | 0.613 |
| Refinement | | | | | |
| *R*[*F*^2^>2*σ*(*F*^2^)] | 0.0280 | 0.0226 | 0.0247 | 0.0221 | 0.0220 |
| *ωR* (*F*^2^) | 0.0748 | 0.0569 | 0.0620 | 0.0546 | 0.0461 |
| *S* | 1.026 | 1.080 | 1.040 | 0.994 | 0.948 |
| No. of reflections | 812 | 826 | 834 | 843 | 843 |
| No. of parameters | 34 | 34 | 34 | 34 | 34 |
| No. of restraints | 0 | 0 | 0 | 0 | 0 |
| H-atom treatment | H-atom parameters constrained | | | | |
| *Δρ*_max_ , *Δρ*_min_ (e Å^-3^) | 0.152, −0.133 | 0.113, −0.119 | 0.094, −0.146 | 0.131, −0.094 | 0.064, −0. 079 |

**Table S3**. Complete data of SC-XRD of the SC-ZIF-8 (sample No.1) collected with decreasing temperature.

| Temperature (K) | 400(2) | 300(2) | 200(2) | 100(2) |
| --- | --- | --- | --- | --- |
| Chemical data | | | | |
| Chemical formula | C_2_H_2.50_NZn_0.25_ | | | |
| *M*_r_ | 56.89 | | | |
| Crystal system | cubic | | | |
| Space group | *I*$\bar{4}$3*m* | | | |
| *a* (Å) | 17.0523(3) | 17.0074(4) | 16.9827(2) | 16.9466(3) |
| *V* (Å^3^) | 4958.5(3) | 4919.4(3) | 4898.02(17) | 4866.8(3) |
| *Z* | 48 | 48 | 48 | 48 |
| *μ* (mm^−1^) | 1.865 | 1.880 | 1.888 | 1.900 |
| crystal size (mm) | 0.1×0.1×0.1 | | | |
| Data collection | | | | |
| Diffractometer | XtaLAB Synergy-S (Rigaku) | | | |
| Absorption correction | multi-scan (CrysAlisPro (Rigaku Oxford Diffraction, 2021)) | | | |
| *T*_min_ | 0.84512 | 0.76595 | 0.76652 | 0.74584 |
| *T*_max_ | 1.00000 | 1.00000 | 1.00000 | 1.00000 |
| No. of measured, independent and observed [*I*>2*σ*(*I*)] reflections | 2828, 841, 774 | 2747, 831, 798 | 2756, 829, 814 | 2721, 824, 796 |
| *R*_int_ | 0.0293 | 0.0280 | 0.0257 | 0.0252 |
| (*sinθ*/*λ*)max (Å^−1^) | 0.615 | 0.615 | 0.613 | 0.615 |
| Refinement | | | | |
| *R*[*F*^2^>2*σ*(*F*^2^)] | 0.0273 | 0.0257 | 0.0171 | 0.0256 |
| *ωR* (*F*^2^) | 0.0700 | 0.0673 | 0.0410 | 0.0635 |
| *S* | 1.014 | 1.059 | 0.994 | 1.033 |
| No. of reflections | 841 | 831 | 829 | 824 |
| No. of parameters | 34 | 34 | 34 | 34 |
| No. of restraints | 0 | 0 | 0 | 0 |
| H-atom treatment | H-atom parameters constrained | | | |
| *Δρ*_max_ , *Δρ*_min_ (e Å^−3^) | 0.095, −0.163 | 0.124, −0.130 | 0.106, −0.110 | 0.123, −0.214 |

**Table S4**. Selected bond lengths (Å) and angles ($^{\circ}$) for ZIF-8 (sample No.1) at different temperature. Atom numbers correspond to those in Figure S2.

| Temperature (K) | 100(2) | 200(2) | 300(2) | 400(2) | 500(2) |
| --- | --- | --- | --- | --- | --- |
| Type of lengths | bond lengths (Å) | | | | |
| Zn1-N2 | 1.987(3) | 1.984(2) | 1.995(3) | 2.001(3) | 1.996(3) |
| C5⋯C5’ | 4.383(11) | 4.361(7) | 4.273(9) | 4.278(11) | 4.287(7) |
| N2’’⋯ N2’’’ | 3.242(6) | 3.243(5) | 3.264(5) | 3.279(6) | 3.263(5) |
| Zn⋯Zn | 5.97297(11) | 5.99358(7) | 6.02126(15) | 6.03993(11) | 6.04746(15) |
| Type of Angles | bond angles ($^{\circ}$) | | | | |
| N2’’-Zn1-N2’’’ | 109.39(16) | 109.61(12) | 109.78(14) | 110.00(15) | 109.67(13) |
| C3-N2’’-C4 | 104.7(4) | 104.6(3) | 103.9(4) | 104.2(4) | 103.7(3) |

**Table S5**. Selected bond lengths (Å) and angles ($^{\circ}$) for ZIF-8 (sample No.1) with decreasing temperature. Atom numbers correspond to those in Figure S2.

| Temperature (K) | 400(2) | 300(2) | 200(2) | 100(2) |
| --- | --- | --- | --- | --- |
| Type of lengths | bond lengths (Å) | | | |
| Zn1-N2 | 1.994(3) | 1.988(3) | 1.9879(17) | 1.988(3) |
| C5⋯C5’ | 4.292(11) | 4.285(9) | 4.310(6) | 4.319(7) |
| N2’’⋯ N2’’’ | 3.261(6) | 3.249(6) | 3.254(4) | 3.252(5) |
| Zn⋯Zn | 6.02890(11) | 6.01302(15) | 6.00429(7) | 5.99153(11) |
| Type of Angles | bond angles ($^{\circ}$) | | | |
| N2’’-Zn1-N2’’’ | 109.73(15) | 109.56(15) | 109.85(10) | 109.78(13) |
| C3-N2’’-C4 | 103.9(4) | 104.3(4) | 104.6(3) | 105.0(3) |

**Notes and references**

[1] O. Karagiaridi, M. B. Lalonde, W. Bury, A. A. Sarjeant, O. K. Farha and J. T. Hupp, J Am Chem Soc, 2012, 134, 18790–18796.

[2] C. Chen, A. Ozcan, A. O. Yazaydin and B. P. Ladewig, J Memb Sci, 2019, 575, 209–216.

[3] C. Zhang, R. P. Lively, K. Zhang, J. R. Johnson, O. Karvan and W. J. Koros, J Phys Chem Lett, 2012, 3, 2130–2134.

[4] C. Chmelik, H. Bux, J. Caro, L. Heinke, F. Hibbe, T. Titze and J. Kärger, Phys Rev Lett, 2010, 104, 085902.

[5] K. S. Park, Z. Ni, A. P. Côté, J. Y. Choi, R. Huang, F. J. Uribe-Romo, H. K. Chae, M. O’Keeffe and O. M. Yaghi, Proc Natl Acad Sci U S A, 2006, 103, 10186–10191.
